# Atmospheric pollen concentrations and chronic obstructive pulmonary disease (COPD) patients visits in Beijing: time series analysis using a generalized additive model

**DOI:** 10.1038/s41598-024-54055-3

**Published:** 2024-02-12

**Authors:** Aizhu Liu, Weixuan Sheng, Xianshi Tang

**Affiliations:** 1 Department of Otolaryngology Head and Neck Surgery, Capital Medical University Affiliated Beijing Shijitan Hospital, Beijing, 100038 China; 2 Department of Anesthesiology, Capital Medical University Affiliated Beijing Shijitan Hospital, Beijing, 100038 China; 3 Key Laboratory of National Health Commission on Parasitic Disease Control and Prevention, Key Laboratory of Jiangsu Province on Parasite and Vector Control Technology, Jiangsu Institute of Parasitic Diseases, Wuxi, 214064 China

**Keywords:** Chronic obstructive pulmonary disease, Environmental meteorological factors, Pollen, Generalized additive model, Time series analysis, Climate sciences, Ecology, Environmental sciences

## Abstract

To investigate the correlation between the daily visits of chronic obstructive pulmonary disease (COPD) patients in hospital clinic and pollen concentrations in Beijing. We collected daily visits of COPD patients of Beijing Shijitan Hospital from April 1st, 2019 to September 30th, 2019. The relationship between pollen concentrations and COPD patient number was analyzed with meteorological factors, time trend, day of the week effect and holiday effect being controlled by the generalized additive model of time series analysis. R4.1.2 software was applied to generate Spearman correlation coefficient, specific and incremental cumulative effect curves of relative risks as well as the response and three-dimensional diagrams for the exposure lag effect prediction. The fitting models were used to predict the lag relative risk and 95% confidence intervals for specific and incremental cumulative effects of specific pollen concentrations. The number of COPD patients was positively correlated with pollen concentration. When pollen concentration increased by 10 grains/1000 mm^2^, the peak value of the specific cumulative effect appeared on day0, with the effect gone on day4 and a lag time of 4 days observed, whereas the incremental cumulative effect’s peak value was shown on day17, and the effect disappeared on day18, with a lag time of 18 days. The results showed that pollen concentration was not only positively correlated with the number of COPD patients, but also had a bimodal lag effect on COPD visits in the hospital at Beijing.

## Introduction

COPD is a chronic respiratory disease characterized by obstructive ventilation disorders, and in most cases it would irreversibly develop into chronic respiratory failure eventually^1^. As a global epidemic with the increasing incidence as high as 10% of the total population over 45 years old^2^, COPD is severely reducing patients’ life quality and work ability and resulting in huge economic losses and health expenditure^3,4^. Surveys from nine Asia–Pacific regions showed that the prevalence of COPD was 6.2% in these areas, with 46% of the patients suffering from exacerbation at least once in the previous year and 19% of them in need of hospitalization^5^. Due to its acute exacerbation as one major reason for the increased mortality, COPD was the third leading death cause and fifth disability cause globally by 2020 and is growing to be the third leading death cause by 2030^6^. In Hong Kong, COPD is the third death-caused respiratory disease, just behind respiratory infections and malignant tumors^7^. A Japanese study on COPD as the main investigated disease found that higher mortality tended to occur in the aged male patients of lower body mass index^8^.

Airborne allergens contain many subcategories, all of which can cause allergic reactions. For example, allergenic pollen can cause increased respiratory symptoms in patients with pre-existing chronic respiratory conditions, often requiring medical intervention for relief. Different allergens have different effects on specific individuals. Based on environmental and clinical data, this manuscript focuses on the effects of pollen on patients with COPD. The extent of pollen’s influence on respiratory diseases varies on climatic zone, vegetation distribution, other regional conditions and patients’ characteristics, and the relevance has not been quantitatively investigated in previous studies^9^. Health effects of the same study population exposed in different conditions could be observed via time series analysis which is commonly used to study environmental factors’ short-term health effects as an internationally accepted standard method^10^. In the analysis, compared with the total population, the patients’ daily visits were seen as events with low probabilities and thus were regarded as a statistic process approximately obeying Poisson distribution, whereas data in terms of meteorology, pollen concentration and outpatients were listed as time series data.

Because environmental exposure factors have lag effects on health outcomes and the exposure factors and responses are non-linearly correlated, the additional time dimension and nonlinear statistical model would be required. Distributed lag non-linear model (DLNM) is a modeling framework that simultaneously describes the nonlinear expose-response relationship and delay effect^11^. Based on the cross basis function using the two-dimensional space of the function to describe the relationship between the predicted value and the lag dimension, DLNM can well represent a variety of exposure–response relationships^12^.

In this paper, a generalized additive model with nonlinear lag time series was used to analyze the influence of atmospheric pollen concentration on COPD visits in Beijing, and was expected to preliminarily predict the influence of environmental pathogens on the incidence of COPD.

## Data and methods

### Data sources

#### Clinical data

According to codes from the 10th Edition of International Standard Diseases Classification (ICD-10), search for the following diagnosis in Beijing Shijitan Hospital Information Center: Obstructive pneumonia, obstructive emphysema, chronic obstructive bronchitis with infection, chronic obstructive pulmonary disease with acute lower respiratory tract infection, chronic obstructive pulmonary disease with acute exacerbation, chronic obstructive bronchitis, chronic obstructive pulmonary disease, chronic obstructive bronchitis, and the data of daily medical visits of diseases listed above were collected from April 1st, 2019 to September 30th, 2019.

#### Environmental data

Meteorological data in the same period collected from the Beijing Meteorological Bureau included the average daily temperature, the dew point, the average relative humidity, the average wind speed, the average air pressure, the total precipitation and pollen concentration.

The Beijing Meteorological Bureau cooperates with Beijing Tongren Hospital affiliated to Capital Medical University to provide pollen concentration. Pollen sampling and taxonomic counting methods: pollen sampling used a Durham instrument by gravity sedimentation, the samplers were installed on the roof top of the outpatient building of Beijing Tongren Hospital, which was 16 m from the floor, the sampling slides were replaced at timed intervals every 24 h, Stained, examined microscopically and counted, the number of all pollen grains under the cover slide of 22 mm × 22 mm was read and converted into the number of pollen grains on the cross-sectional area of 1000 mm^2^, namely the pollen content. The unit is grains/1000 mm^2^.

### Statistical analysis

Spearman correlation coefficient was generated via R4.1.2 software, and the RR value of influencing factors on COPD incidence was calculated by DLNM packet fitting models. The lag response curves of RR’s specific and incremental cumulative effect and the response and three-dimensional graphs for exposure lag effect prediction were all plotted to summarize the relationship between pollen concentration parameters and COPD patient daily visits. The fitting models were used to predict the lag relative risk (RR) and 95% confidence intervals (CI) for specific and incremental cumulative effects of specific pollen concentrations.

### Model construction.

The basic formula of Generalized Additive Model (GAM) used in the study is:

$${{\it Yt}}\sim {\it{Poisson}}(\mu {\it{t}})$$

$$\mathrm{log }\mu =\beta 0+\beta \mathrm{ \,cb}.Xt+\mathrm{ s}\left({\text{t}}, d{\text{f}}\right)+{\text{s}}\left(Zt, d{\text{f}}\right){\text{DOW}}.$$

*Yt* is the number of visits for COPD at day t; μ is the expected number of visits for COPD at day t; β0 is the intercept; β is the vector of coefficients for cb.*Xt*; t is the time variable; cb.*Xt* is the cross-basis of atmospheric pollen concentration at day t; s is the spline smoothing function; *Zt* is the meteorological factor; df is the degree of freedom; DOW is the day of the week and public holidays represented as categorical variables, using to control short-term fluctuations.

As the health effect evaluation model commonly used in epidemiology, GAM is applicable and effective for nonlinear regression analysis of health effects of environmental factors such as air temperature and pollutants^13^, for its flexibility in explaining the dependence on covariates and the simplicity in specifying model based on “smoothing function” instead of complicated parameter relations. The models were successively fitted and the degree of freedom (df) of the time smoothing function was determined as the minimum df value corresponding to the sum of the Partial autocorrelation function (PACF)’s absolute residual value when the maximum lag time 30d was chosen. The natural spline smoothing function was used to avoid the confounding effect of meteorological factors. According to the former studies, the df value of the environmental factors related model was set at 3^14^, and after introducing the allergens cross-base to establish the basic model, the influence on the COPD patient number was analyzed. AIC (Akaike information criterion) simultaneously reflecting goodness of fit and number of independent variables was adopted in the study for its wide application in autoregression order determination in time series analysis, independent variables screening in multiple regression and generalized linear regression, etc. The smaller the AIC value showed, the better goodness of fit of the model was obtained^15^. The RR value is between 0 and ∞. RR = 1, indicating no correlation between exposure and disease; RR < 1, indicating a decrease in disease incidence caused by exposure; RR > 1, indicating that exposure causes additional morbidity as a risk factor. The exposure–response relationship coefficient β was estimated by the GAM model and when the pollen concentration changed by unit Δc, the RR representing relative change of the natural logarithm of COPD population and its 95% confidence interval (CI) were calculated as follows: ${\text{RR}}={\text{exp}}\left(\upbeta +\Delta \complement \right),\mathrm{ RR }95\mathrm{\% CI}={\text{exp}}[(\upbeta \pm 1.96{\text{SE}})\times \Delta \complement ].$ SE is the standard deviation of β^16^.

### Ethical approval

This clinical study is a retrospective study. Clinical data of patients are only collected without intervention in the treatment plan of patients, so there is no risk to patients' physiology. The researchers will do their best to protect the information provided by patients as personal privacy from being leaked without informed consent.

### Consent to participate

In this retrospective study, only the number of patients was collected, no personal privacy was involved, and no informed consent was obtained.

## Results

### Statistical description of meteorological variables, pollen concentration and patient number (April 1st, 2019–September 30th, 2019)

**Figure 1:**
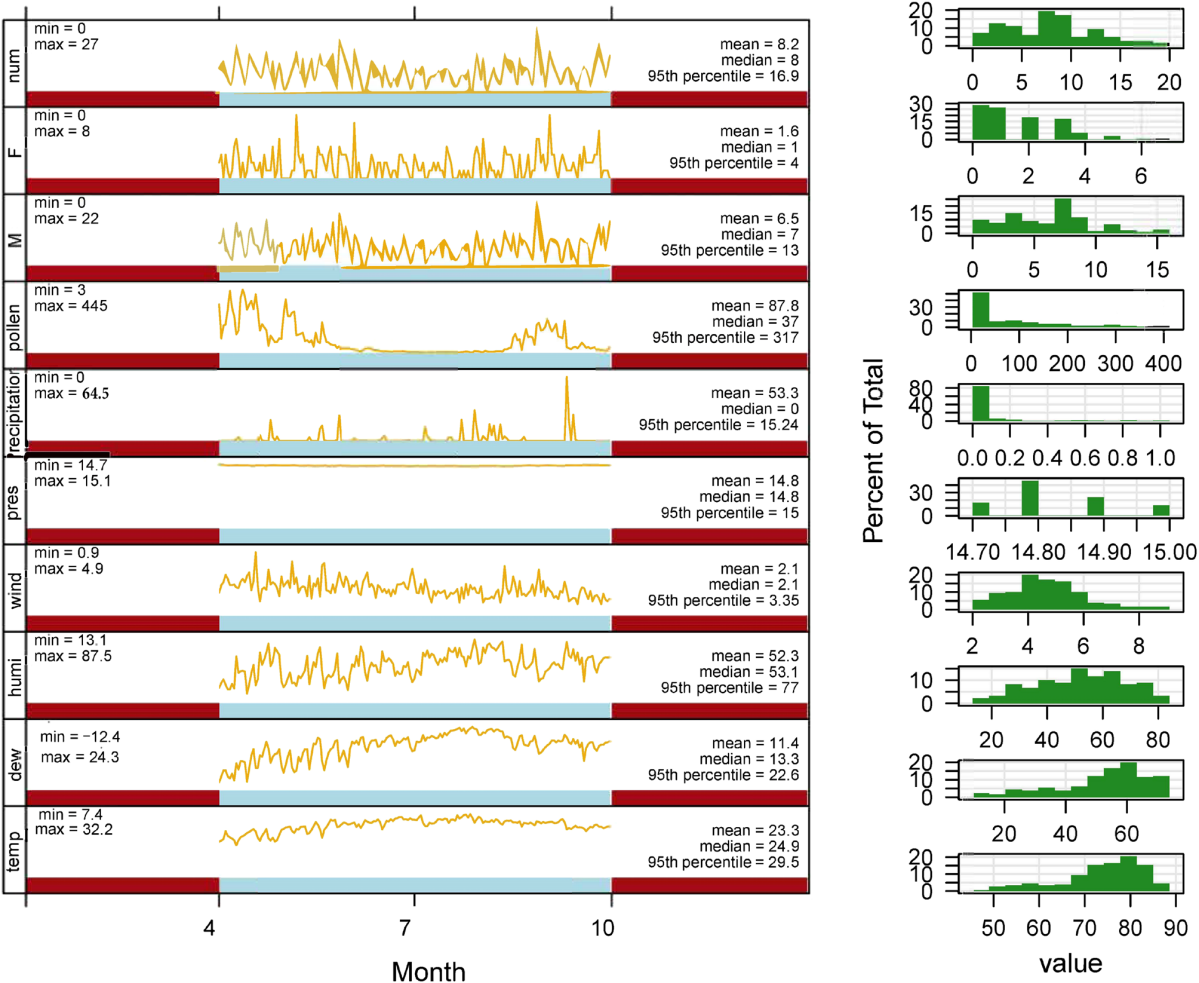
Time series diagram of the daily patient number, meteorological variables and pollen concentration.

The statistical values of meteorological variables considered in this study were listed in Table 1 including temperature, dew, relative humidity, wind speed, air pressure, precipitation and pollen concentration. The statistic analysis via Spearman correlation in Table 2 showed that the mean temperature (−0.187, *P* < 0.05), the dew point (−0.244, *P* < 0.01) and relative humidity (−0.184, *P* < 0.05) were negatively related to the COPD patient visits, whereas the mean atmospheric pressure (0.171, *P* < 0.05) and atmospheric pollen concentration (0.234, *P* < 0.01) were positively-related factors. Different from the other variables and COPD visits that did not show apparent distribution pattern throughout the months in Fig. 1, pollen concentration were of a significant seasonal distribution trend, with the minimal amount in summer and the concentration peaks in spring and autumn, specifically in April, May and September of the year.

**Table 1:** Statistical description of daily meteorological variables, pollen concentration and patient number (n = 183).

	Unit	Mean value	Median	SD (standard deviation)	Minimum	Maximum	Percentiles	
							25	75
Temperature	℃	23.3	24.9	3.744	7.4	32.2	20.7	26.9
Dew	℃	11.4	13.3	4.567 8	−12.4	24.3	7.2	17.2
Humidity	%	52.3	53.1	16.773 1	13.1	87.5	40.1	64.5
Wind Speed	m/s	2.1	2.1	0.913 4	0.9	4.9	1.7	2.5
Air Pressure	mmHg	14.8	14.8	0.098 3	14.7	15.1	14.8	14.9
Precipitation	mm	53.3	0	1.578	0	64.5	0	0.25
Pollen	grains/1000 mm^2^	87.8	37	105.596	3	445	10	131
Male		6.5	7.0	3.898	0	22	4	9
Female		1.6	1	1.593	0	8	0	3
Total number of COPD patients		8.2	8	4.868	0	27	5	11

**Table 2:** Spearman correlation coefficients of meteorological variables, pollen concentration and patient number.

	Temperature	Dew	Humidity	Wind	Press	Precipitation	Pollen	Number of COPD patients
Temperature	1.000	0.681**	0.156*	−0.142	−0.716**	−0.141	−0.649**	−0.187*
Dew		1.000	0.780**	−0.462**	−0.578**	0.220**	−0.731**	−0.244**
Humidity			1.000	−0.529**	−0.206**	0.440**	−0.509**	−0.184*
Wind				1.000	−0.018	−0.111	0.318**	0.085
Press					1.000	−0.063	0.550**	0.171*
Precipitation						1.000	−0.221**	0.004
Pollen							1.000	0.234**
Number of COPD patients								1.000

*P < 0.05, **P < 0.01.

### The Lag analysis (specific and incremental cumulative effect) of pollen concentration on COPD visits

**Figure 2:**
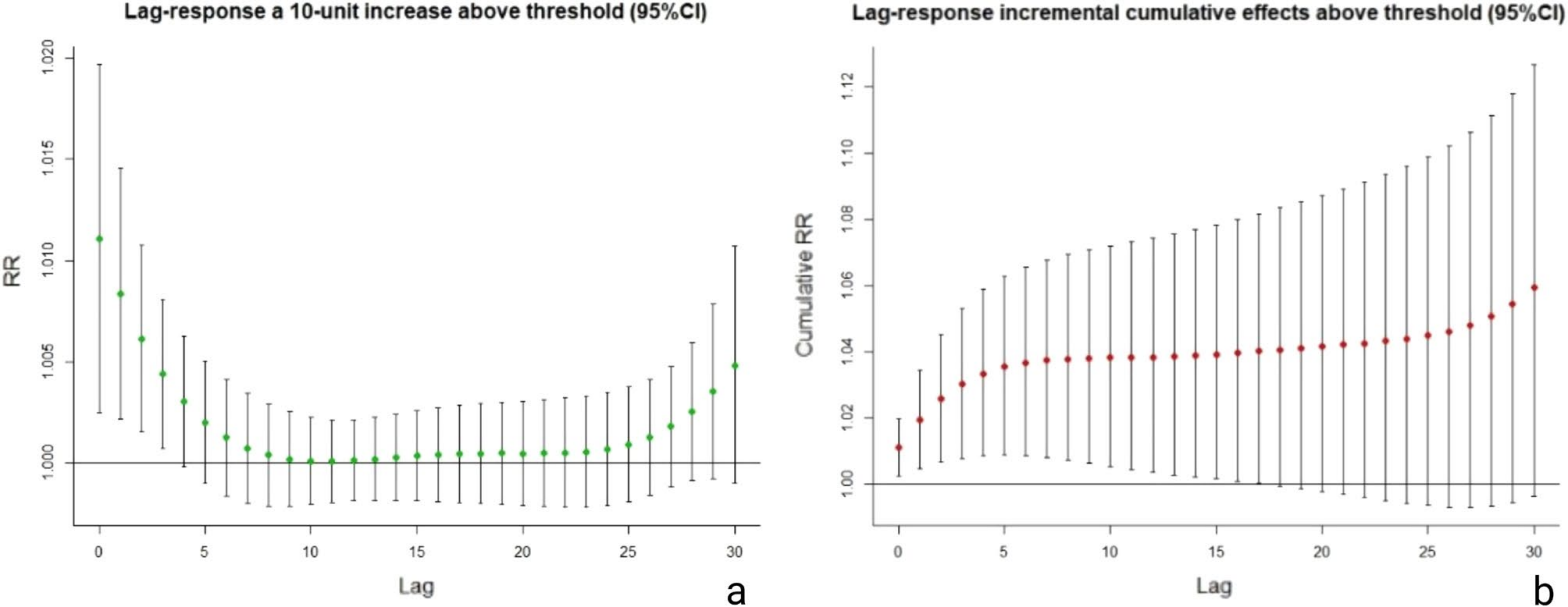
**(a)** The response curve of specific lag effects and the corresponding RR curve for every additional 10 grains/1000 mm^2^ of pollen. **(b**) The response curve of incremental cumulative lag effects and the corresponding RR curve for every additional 10 grains/1000 mm^2^ of pollen.

**Figure 3:**
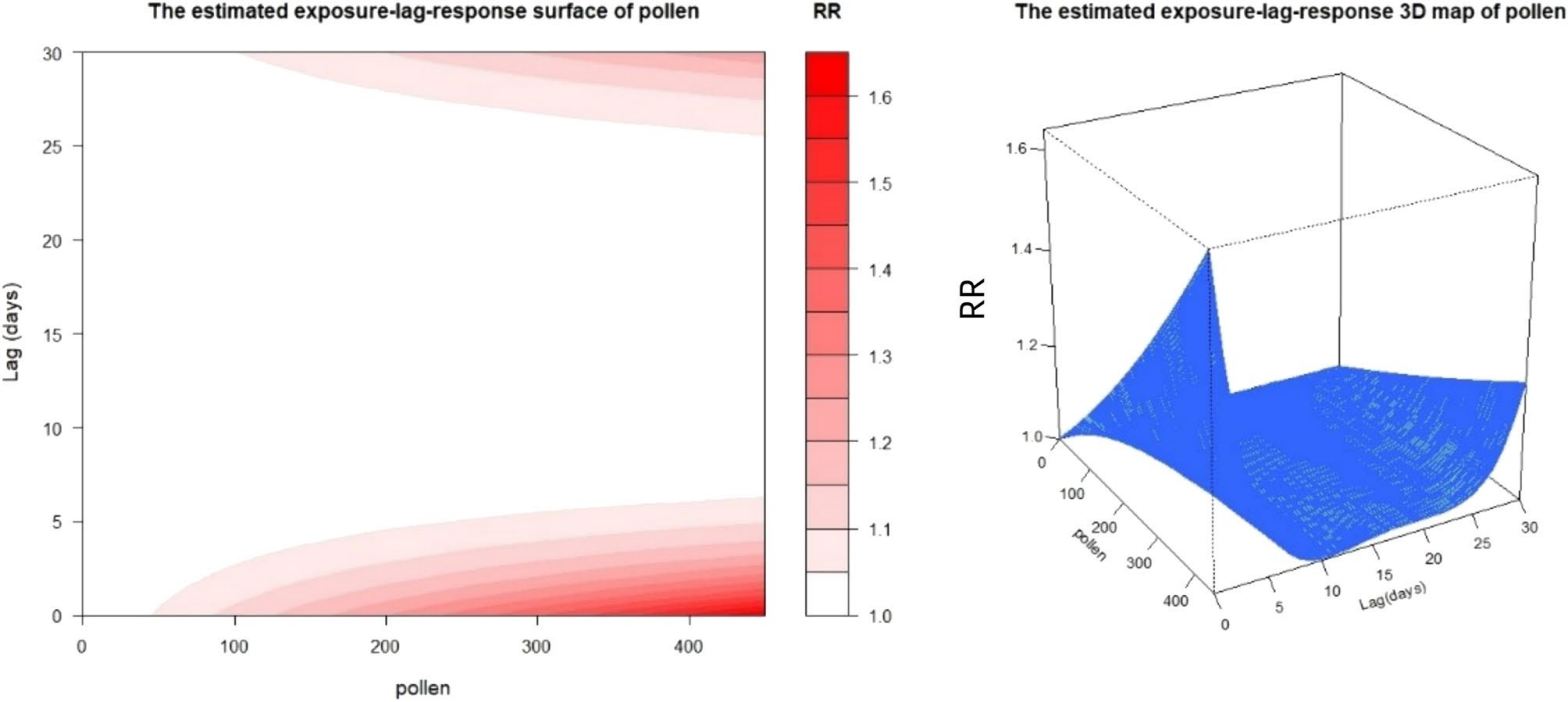
The response diagram and three-dimensional diagram of estimated expose lag response of pollen (R^2^ = 0.517).

When the atmospheric pollen concentration was increased by 10 grains/1000 mm^2^, the specific cumulative effect reached its peak value on day0 and disappeared on day4, with a lag time of 4 days (Fig. 2a), whereas the peak value of the incremental cumulative effect was shown on day17 and the effect fell into disappearance on day18, thus its lag time was 18 days (Fig. 2b). As pollen concentration increased, the more apparent and lasting lag effect was shown in the diagram (Fig. 3). And the lag effect of pollen concentration on COPD visit number exhibited a double-peak phenomenon with no lag effects before day 5 and after day 25 in the condition of the atmospheric pollen concentration being less than 50 grains/1000 mm^2^.

**Figure 4:**
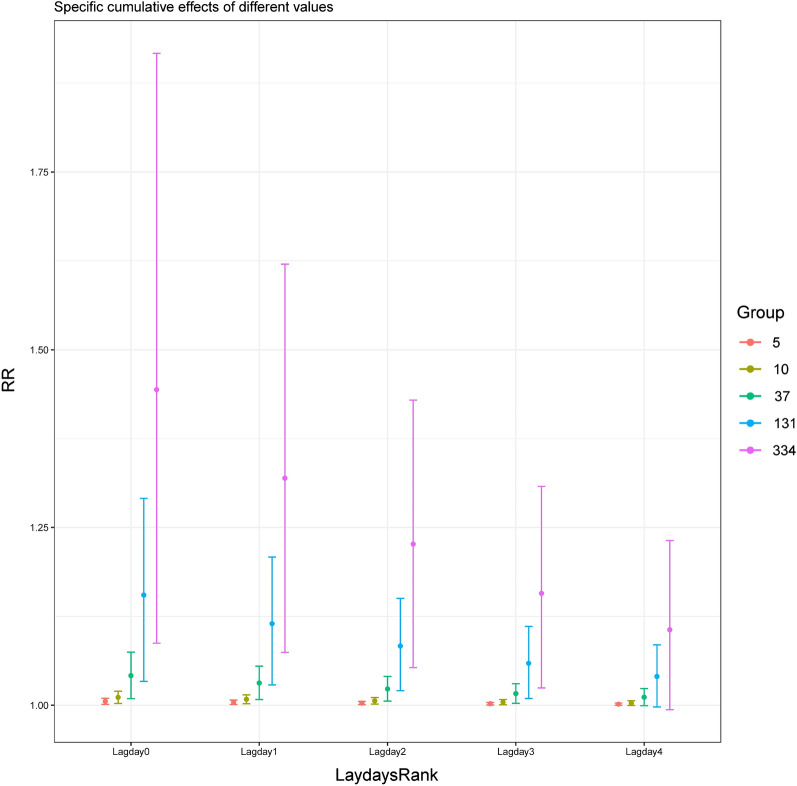
The specific cumulative lag RR value matrix and 95% confidence interval for P5, P25, P50, P75, and P95.

**Figure 5:**
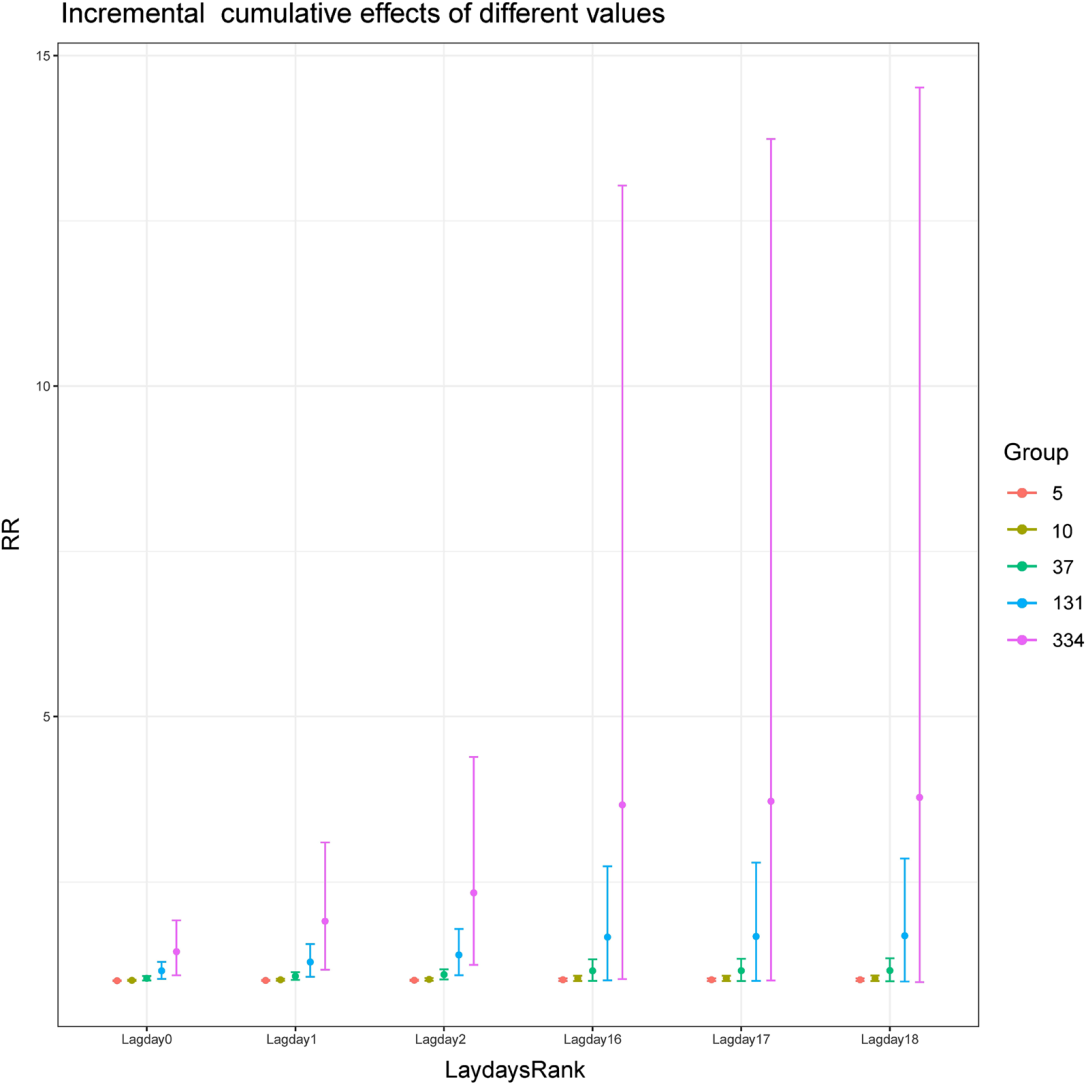
The incremental cumulative lag RR matrix and 95% confidence interval for P5, P25, P50, P75, and P95.

GAM was used to obtain the RR value matrix and their 95% confidence intervals of pollen concentration cumulative lag effect at specific exposure values. Figure 4 showed that using the established GAM model to calculate the specific cumulative lag RR value matrix and 95% confidence interval for P5 (5 grains/1000 mm^2^), P25 (10 grains/1000 mm^2^), P50 (37 grains/1000 mm^2^), P75 (131 grains/1000 mm^2^), and P95 (334 grains/1000 mm^2^), and plot them. The peak values of P5, P25, P50, P75 and P95 specific cumulative effect (Fig. 4) were all shown on day0, and the corresponding RR was respectively 1.006 (95% CI: 1.001, 1.010), 1.011 (95% CI: 1.003, 1.020), 1.042 (95% CI: 1.009, 1.075), 1.155 (95% CI: 1.033, 1.291), 1.444 (95% CI: 1.087, 1.917), with the RR values of P5, P25, P50, P75 and P95 of no statistical significance on day4, and thus the lag time of 4 days. Figure 5 showed that using the established GAM model to calculate the incremental cumulative lag RR value matrix and 95% confidence interval for P5 (5 grains/1000 mm^2^), P25 (10 grains/1000 mm^2^), P50 (37 grains/1000 mm^2^), P75 (131 grains/1000 mm^2^), and P95 (334 grains/1000 mm^2^), and plot them. whereas the peak values of P5, P25, P50, P75 and P95 incremental cumulative effect (Fig. 5) occurred on day17, and the corresponding RR was respectively 1.020 (95% CI: 1.000, 1.040), 1.040 (95% CI: 1.000, 1.082) and 1.157 (95% CI: 1.001, 1.337), 1.674 (95% CI: 1.003, 2.794), 3.720 (95% CI: 1.008, 13.737). The incremental cumulative effect RR values of P5, P25, P50, P75 and P95 showed no statistical significance on day18, and the lag time was 18 days. RR value and 95%CI were shown in the diagram, and reference value is: pollen concentration = 0.

## Discussion

COPD is characterized by the major pathological changes including fibrosis-caused small airway stenosis and lung parenchymal elasticity loss leading to peripheral airway collapse. The inflammation is mainly confined to the peripheral airway wall and lung parenchyma and manifested by the marked increase of neutrophils, macrophages as well as T and B lymphocytes^17^. Most cases of COPD exacerbation are caused by respiratory viral infections, but it has been proven that air pollutants and allergens would aggravate the disease^18^. The COPD onset of nearly 50% patients is triggered by at least 1 inducing factor, and non-infectious factors have been reported more frequently than infectious ones, for COPD patients are sensitive to many environmental factors^19^.

In general, the allergen combined with particles smaller than 5 μm can be suspended in the air for a longer time^20^ , it pass through the airway and deposit in the pulmonary alveoli and central alveolar regions, where they induce allergic reactions consisting of the sensitizing stage including cytokines IL-4, IL-5 and IL-13 secretion by Th2 cells and specific IgE production and the effector stage characterized by the degranulation of mast cells and basophils and the release of mediators (e.g., histamine, prostaglandin, leukotrienes). Allergic patients with COPD sensitized by pollen subsequently develop airway hyperreactivity^21^, which is followed by the onset of COPD. Allergens also induce abnormal immune responses by causing airway epithelial barrier dysfunction, and disruption of epithelial connections may regulate signaling pathways involved in differentiation, recovery and pro-inflammatory responses, increasing the likelihood of COPD^22^. In addition, there are multiple associations between allergen exposure and upper and lower respiratory tract inflammation^23^. The incidence of allergic diseases induced by pollen is thus predicted to increase in the medium to long term^24^.

At present, few studies have been conducted on the effects of air-borne pollen on COPD^25^. Although allergization has been commonly considered in the studies of chronic respiratory diseases including asthma, COPD and bronchiectasis^26^, it is generally seen as a whole instead of being further quantitatively analyzed^27^. In this study, we focused on air-borne pollen from multiple allergenic factors and investigated the pollen’s allergenic effects on COPD via statistic methods.

The pollen distribution in Beijing urban area presents a bimodal pattern throughout the year, with the spring peak mainly resulting from tree pollen during March and April and the autumn peak of grass pollen from late August to late September. The air-borne pollen in spring and autumn are more allergenic than the summer insect-borne pollen. Pollen sickness in Beijing is mainly caused by the pollen of highly allergenic herbaceous plants, whose pollen period is concentrated in spring and autumn^28^. Similarly, there are also two peaks of air-borne pollen concentration within one day, one around 02:00 am and the other at about 14:00 pm, whereas the lowest level is around 22:00 pm. Factors influencing pollen distribution include weather conditions, such as wind pressure, wind speed, wind direction, precipitation, humidity, etc., as well as microscopic and macroscopic topography of the studied region^29^. While sunlight and high temperature promote pollen maturation, the rainfall does the opposite. When the relative humidity is between 20 and 50% or greater than 70%, lowered humidity serves to dry pollen grains, making them more ready to release, and when the relative humidity is between 50 and 60%, the air-borne pollen concentration correlates positively with the relative humidity. Pollen tend to be far spread at the wind speed of 1–3 m/s, and then the air-borne pollen content goes up; In contrast, if the wind speed is over 4 m/s or lasts for long, the pollen content in the air turns out to be declining^30^. Allergen exposure depends not only on their environmental distribution, but also on their air-borne transmission form and aerodynamic characteristics^31^. Allergens released from pollen grains into the air give rise to a type of super-fine aerosol. And the pollen are gaining increased allergenicity as global warming elongates and intensifies flowering. Previous data have shown that air allergens are growing more powerful on allergic patients, increasing the possibility of allergic respiratory diseases onset and aggravating the condition of patients with symptoms^32^.

In this study, we showed that neither the total number of COPD patient visits nor the number of male or female patients was of significant seasonal trends (Table 1 and Fig. 1), suggesting that environmental factors including meteorological conditions and the air-borne pollen may have mixed effects on COPD, some acting inducing, others inhibiting, and their effects may cancel each other out. Although the correlation analysis between atmospheric pollen concentration and other environmental factors was not conducted in this study to assess the impact of these factors on the association between pollen rise and increased COPD visits, compared with other environmental factors, the correlation coefficient between pollen concentration and the number of COPD patients visit was in the relatively high level (Table 2).

The lag effect calculation showed that for every 10 grains/1000 mm^2^ increase in pollen concentration in 2019, the specific cumulative effect had a lag time of merely 4 days, whereas the lag time of the incremental cumulative effect was as long as 18 days (Fig. 2a, b), which proved that the elevated air-borne pollen level had both short-termed inducing effect and medium to long-term cumulative effects on COPD, and the higher pollen level went, the longer and stronger their lag effect became. Besides, at a low concentration of 50 grains/1000mm^2^, the lag effect of pollen concentration on COPD visit number not only existed, but also exhibited a double-peak phenomenon with no lag effects before day 5 and after day 25 (Fig. 3), which meant that when the pollen concentration reached the threshold, type I hypersensitivity reaction was started on the same day and patients showed clinical symptoms. Finally, we used the established GAM model to predict the RR value and 95% CI of the specific cumulative effect of different pollen exposure concentrations, and the RR value and 95%CI of the incremental cumulative effect, and visualized them, as shown in Figs. 4 and 5. So that timely predictions could be made according to different pollen concentrations in clinical work.

## Conclusion

In the present study, we have confirmed through modeling and calculation that pollen concentration has an impact on the onset of COPD. Since it is difficult to distinguish pollen types at peak pollen concentration in different time periods in this region, we took atmospheric pollen concentration as a whole to explore and analyze its relationship with COPD incidence in this study. Follow-up research directions would include multi-center research, studies covering more than 1 year, and age and gender stratification analysis for all COPD patients, so as to obtain a deeper and more comprehensive understanding and more accurate prediction of the impact of pollen concentration on COPD in Beijing area.

## Data Availability

The datasets generated during and/or analysed during the current study are available from the corresponding author on reasonable request.
